# Heat-Treated Meat Origin Tracing and Authenticity through a Practical Multiplex Polymerase Chain Reaction Approach

**DOI:** 10.3390/nu14224727

**Published:** 2022-11-09

**Authors:** Yan Cheng, Sha Wang, Shilong Ju, Song Zhou, Xiaoqun Zeng, Zhen Wu, Daodong Pan, Guowei Zhong, Zhendong Cai

**Affiliations:** 1 Key Laboratory of Animal Protein Deep Processing Technology of Zhejiang Province, College of Food and Pharmaceutical Sciences, Ningbo University, Ningbo 315800, China; 2 Key Laboratory of Vector Biology and Pathogen Control of Zhejiang Province, Huzhou University, Huzhou 313000, China; 3 Center for Global Health, School of Public Health, Nanjing Medical University, Nanjing 211166, China

**Keywords:** molecular traceability, multiplex PCR technique, meat species, species-specific primer, meat adulteration

## Abstract

Meat adulteration have become a global issue, which has increasingly raised concerns due to not only economic losses and religious issues, but also public safety and its negative effects on human health. Using optimal primers for seven target species, a multiplex PCR method was developed for the molecular authentication of camel, cattle, dog, pig, chicken, sheep and duck in one tube reaction. Species-specific amplification from the premixed total DNA of seven species was corroborated by DNA sequencing. The limit of detection (LOD) is as low as 0.025 ng DNA for the simultaneous identification of seven species in both raw and heat-processed meat or target meat: as little as 0.1% (*w*/*w*) of the total meat weight. This method is strongly reproducible even while exposed to intensively heat-processed meat and meat mixtures, which renders it able to trace meat origins in real-world foodstuffs based on the authenticity assessment of commercial meat samples. Therefore, this method is a powerful tool for the inspection of meat adulterants and has broad application prospects.

## 1. Introduction

Meat contains a variety of micronutrients of rich protein, amino acids, minerals, fats, fatty acids and vitamins essential for growth and development [1]. Meat products are highly popular, as one of the most favored and consumed foods worldwide. With economic growth, the demand for meat products continuously increases, also causing the increased incidence of meat deception [2]. Therefore, meat authenticity has become a hot topic based on various concerns, including religious affairs, specific meat allergies, malicious marketing practices and economic and legal reasons [3,4]. For example, pork and cattle consumption are strictly forbidden for Islam and Hinduism, respectively [5,6]. In addition, vegetarians and vegans reject meat ingredients. Furthermore, certain people such as sensitized patients are allergic to specific meat species [7]. Nowadays, meat deception has become commonplace, with examples including the most notorious horse meat scandal of the European Union in 2013, which disrupted consumer trust and caused a deleterious effect on the meat industry [8]. It is necessary to develop practical techniques to assess meat authenticity, which is considered an important step to protect consumers from commercial fraud and thereby establish discipline in food industry.

It is worth noting that meat products are often subjected to processing procedures, providing more opportunities for meat adulteration with cheaper or controversial species [9,10,11]. Accordingly, visual discrimination of meat species is complicated and the methodologies have to provide an accurate judgement for meat ingredients in processed meat products [12,13,14,15,16]. To date, various analytical methods such as protein- and DNA-based methods have been constructed and are widely utilized for meat authentication in composite mixtures [17]. Nonetheless, protein-based methods are tissue-dependent and unstable during food processing, which in turn fail to accurately detect polypeptide targets [1]. Unlike proteins, DNA is more stable, existing in not only fresh meat products but also processed ones, which has contributed to DNA analysis as a preferential choice for the identification of meat origins in processed products [18]. From DNA-based techniques, polymerase chain reaction (PCR) including multiplex PCR, real-time PCR and PCR-RFLP has evolved as the practical method for meat species detection under various processing conditions [2,4]. Nevertheless, multiplex PCR systems with species-specific primers are greatly desirable since they can simultaneously inspect multiple meat types without special infrastructures [19].

Nowadays, species-specific PCR amplification targeting mitochondrial genes has gained great attention since there are multiple copies harboring intraspecies conservation and interspecies polymorphism in mitochondrial DNA (mtDNA) [1,4]. Here, using mtDNA genes including NADH dehydrogenase subunit 2, 4 and 5, cytochrome c oxidase subunit I and III, 16S rRNA as targets, primers for seven animal species—camel, cattle, dog, pig, chicken, sheep and duckwere designed and selected through the analyses of cross-reactivity, specificity, sensitivity and robustness. Using the optimized PCR system, a heptaplex PCR technique with high efficiency, sensitivity and accuracy was developed using seven pairs of target primers. This method achieved the simultaneous detection of seven meat species in both raw and processed meat products.

## 2. Materials and Methods

### 2.1. Samples Collection and DNA Extraction

Fresh pure meat and meat products were purchased from local markets in Ningbo City, PR China, as well as on an online supermarket platform. Samples were transported on ice and then stored at −80 °C. Genomic DNA was isolated using a Genomic DNA Mini Preparation Kit with a Spin Column (D0063, Beyotime Biotechnology, Shanghai, China) according to the manufacturer’s instructions. DNA concentration was measured by a NanoDrop 2000 spectrophotometer (NanoDrop 2000, UV–Vis spectrophotometer, USA) [17].

### 2.2. Design of Species-Specific Primers

To discriminate between camel, cattle, dog, pig, chicken, sheep and duck, Oligo 7.0 software and BLAST programs were used for designing target primers according to physical parameters of cross-reactivity, melting temperature, self-complementarity and secondary structures [3]. mtDNA sequences including NADH dehydrogenase subunit 4 of camel (GenBank Accession No. MH109991.1), cytochrome c oxidase subunit I of cattle (MN714195.1), NADH dehydrogenase subunit 5 of dog (MN181404.1), NADH dehydrogenase subunit 4 of pig (KJ746666.1), 16S rRNA of chicken (MK163565.1), NADH dehydrogenase subunit 2 of sheep (KP702285.1) and cytochrome c oxidase subunit III of duck (MK770342.1) were retrieved from the National Centre of Biotechnology Information (NCBI) database. To determine in silico specificity, each primer pair was aligned against land animals of camel (*Camelus bactrianus*), cattle (*Bos taurus*), dog (*Canis lupus*), pig (*Sus scrofa*), chicken (*Gallus gallus*), sheep (*Ovis aries*), duck (*Anas platyrhynchos*)*,* horse (*Equus caballus*), cat (*Felis catus*), pigeon (*Columba livia*), rabbit (*Oryctolagus cuniculus*), ostrich (*Struthio camelus*), turkey (*Meleagris gallopavo*) and goose (*Anser cygnoides*) using a ClustalW sequence alignment program and MEGA6 software. In addition, each primer pair was aligned against three aquatic species of tuna (*Thunnus orientalis*), small yellow croaker (*Larimichthys polyactis*) and black carp (*Mylopharyngodon piceus*). Furthermore, cross reaction was individually checked for target primers using each template from all species aforementioned through simplex PCR assays. Detailed information of primer sets is listed in Table 1. Primers were synthesized by Shanghai Sangon Biological Engineering Technology & Services Co., Ltd. (Shanghai, China).

### 2.3. Simplex and Multiplex PCR Assays

Simplex PCR assays were constructed as follows. Using EasyTaq^®^ DNA Polymerase kit (TransGen Biotech Co., Ltd., Beijing, China), PCR reaction system (25 μL) was constituted by 2.5 μL of 10× EasyTaq^®^ Buffer, 2 μL of 2.5 mM dNTPs, 0.5 μL of 5 units μL^−1^ EasyTaq DNA Polymerase, 0.5 μL of 10 μM forward and reverse primers, target DNA and refilled ddH_2_O to 25 μL. PCR reaction was started at 94 °C for 5 min, followed by 34 cycles of 94 °C for 30 s, 60 °C for 30 s, 72 °C for 45 s, and finally elongated at 72 °C for 5 min. For multiplex PCR assays, PCR reaction system (25 μL) included 2.5 μL of 10× EasyTaq^®^ Buffer, 2 μL of 2.5 mM dNTPs, 0.5 μL of 5 units μL^−1^ EasyTaq DNA Polymerase, 0.5 μL of 10 μM each primer of seven species, target DNA of seven species at the indicated concentration and ddH_2_O. Consistently, PCR reaction was started at 94 °C for 5 min, followed by 34 cycles of 94 °C for 30 s, 60 °C for 30 s, 72 °C for 45 s and finally elongated at 72 °C for 5 min. PCR amplification for both simplex and multiplex PCR assays was performed by T100™ Thermal Cycler (Bio-Rad, Puchheim, Bavaria, Germany). PCR products were electrophoresed on 5% agarose gels by using 4 S GelRed Nucleic Acid Stain, and visualized by Gel Doc^TM^ XR+ System with Image Lab^TM^ Software (BIO-RAD) [23].

### 2.4. Sequencing of PCR Products

PCR fragments were isolated from the agarose gel and purified using DiaSpin DNA Gel Extraction Kit (Shanghai Sangon Biological Engineering Technology & Services Co., Ltd., Shanghai, China). Next, the fragments were cloned into a *pEASY^®^*-T5 zero cloning vector according to the manufacturer’s protocol (TransGen Biotech Co., Ltd., Beijing, China). Using the following plasmid as target template, PCR amplification was performed with vector primers M13F (5′-GTAAAACGACGGCCAGT-3′) and M13R (5′- CAGGAAACAGCTATGAC-3′). PCR products were sequenced by an automated DNA sequencer (Applied Biosystems, Foster City, CA, USA). DNA base composition was analyzed by a BLAST search against the NCBI nucleotide database.

### 2.5. Tests of Specificity, Sensitivity and Reproducibility of Primers

Using the simplex PCR system, the specificity of the target primer was firstly checked against land animals including sheep, dog, horse, chicken, duck, turkey, pigeon, camel, rabbit, ostrich, cattle, cat, goose and pig, as well as aquatic species including small yellow croaker, tuna and black carp. PCR fragments were run on 5% agarose gel and visualized for proper amplification. To examine the sensitivity of the multiplex PCR assay, ten concentrations ranging from 10 to 0.01 ng were selected as the template contents s of multiplex PCR assays. Sensitivity tests were performed using the aforementioned multiplex PCR system. To determine the sensitivity of the model mixtures, the raw meat tissue of camel, dog, pig, chicken, sheep and duck were weighed at 0.1%, 0.25%, 0.5%, 1%, 2.5%, 5%, 10% and 15%, respectively, of the total weight. Next, meat samples weighed at the same proportion were simultaneously added to cattle and homogenized. The limits of detection and dynamic range were determined according to the agarose gel analysis and electropherograms. To determine the reproducibility of species-specific primers, meat tissues were subjected to a heat processing treatment of boiled (97–99 °C, 30 min) and microwave-cooked (750 W, 10 min) patterns, respectively. Primers’ reproducibility was confirmed through PCR amplification using genomic DNA of heat processing samples [24].

## 3. Results

### 3.1. Specificity Assays of Designed Primers

**Figure 1:**
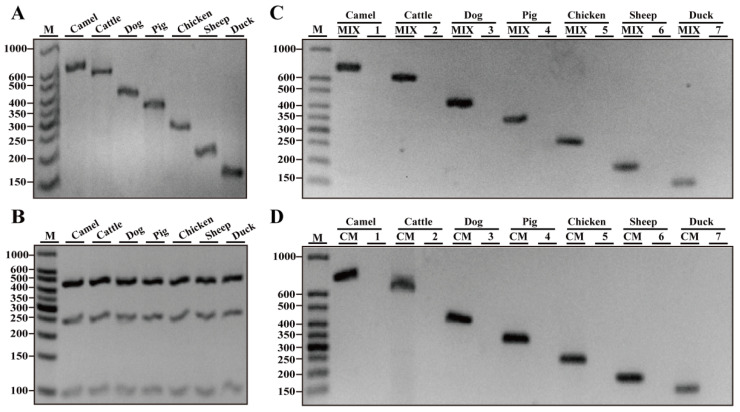
Verification of primer specificity with conventional simplex PCR. (**A**) Simplex PCR detection using species-specific primers for camel, cattle, dog, pig, chicken, sheep and duck origin and respective genomic DNA as the template. (**B**) PCR amplification with premixed universal primers of eukaryotic 12S rRNA, 16S rRNA and 18S rRNA genes for each meat species, respectively. (**C**) PCR amplification using individual template DNA from camel, cattle, dog, pig, chicken, sheep and duck species. MIX, a mixture of seven primer pairs of camel, cattle, dog, pig, chicken and sheep species; 1–7, a mixture of six primer pairs of six nontarget species. (**D**) PCR amplification with a pair of target primers. CM, a complete DNA mixture of seven species including camel, cattle, dog, pig, chicken, sheep and duck; 1–7, DNA mixture of six nontarget species. Lane M is ladder DNA.

To obtain species-specific primers, candidate primers were designed and screened through simplex and multiplex PCR assays. Target primers were ultimately validated and listed in Table 1. Expectedly, target bands with the predicted sizes of 726 bp, 610 bp, 428 bp, 332 bp, 268 bp, 205 bp and 163 bp were amplified for camel, cattle, dog, pig, chicken, sheep and duck (Figure 1A). Next, three universal eukaryotic primer pairs were employed as positive controls to evaluate the DNA quality of meat resources through multiplex PCR amplification. As shown in Figure 1B, target bands of 99 bp, 240 bp and 456 bp had similar intensities existing in all seven meat samples, suggesting that there are identical quality of DNA templates ensuring the accuracy of the subsequent multiplex PCR amplification.

To further examine the specificity of the target primers in this multiplex assay, the genomic DNA of a single species was individually employed for multiplex PCR amplification. They produced the expected targets in the presence of all primer mixtures of seven but not six nontarget primer pairs (Figure 1C). Meanwhile, target primers could specifically amplify target bands in the presence of the DNA mixture isolated from seven but not six nontarget species (Figure 1D). Collectively, it can be postulated that a target primer pair can specifically amplify target species.

To further confirm the accuracy of the PCR amplification, target amplicons in Figure 1D were individually cloned and sequenced. DNA sequencing demonstrated that PCR products amplified by target primers showed an accuracy of 100% based on a BLAST search against the NCBI nucleotide database. In addition, target primers were confirmed without cross-reactivity against sixteen nontarget species through PCR assays (data not shown). Therefore, target primers are highly specific for the inspection of animal species.

### 3.2. Sensitivity of Multiplex PCR Assay

**Figure 2:**
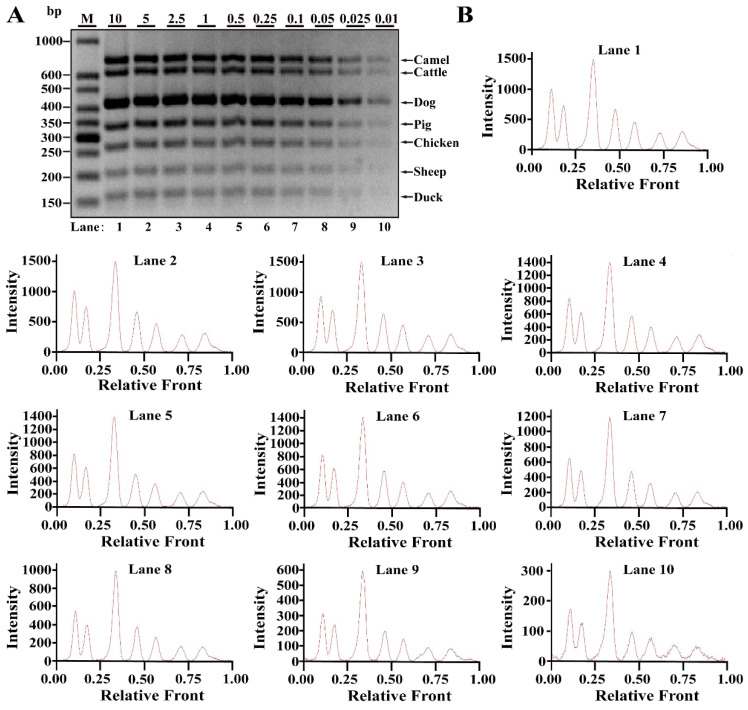
Validation of sensitivity of multiplex PCR assay in raw meat tissues. (**A**) Gel image of PCR fragments amplified by multiplex PCR using premixed DNA contents of seven species (10, 5, 2.5, 1, 0.5, 0.25, 0.1, 0.05, 0.025 and 0.01 ng) with seven sets of species-specific primers, respectively. (**B**) The corresponding electropherograms represent camel, cattle, dog, pig, chicken, sheep and duck in each lane. Lanes 1–10 are presented with labels of 10, 5, 2.5, 1, 0.5, 0.25, 0.1, 0.05, 0.025 and 0.01 in (**A**). The value of number at the horizontal line means the relative position of peaks distant from the top of agarose gel. The value of number at the vertical line means the fluorescent intensity of DNA-bound dyes (4 S GelRed Nucleic Acid Stain). Lane M is ladder DNA.

**Figure 3:**
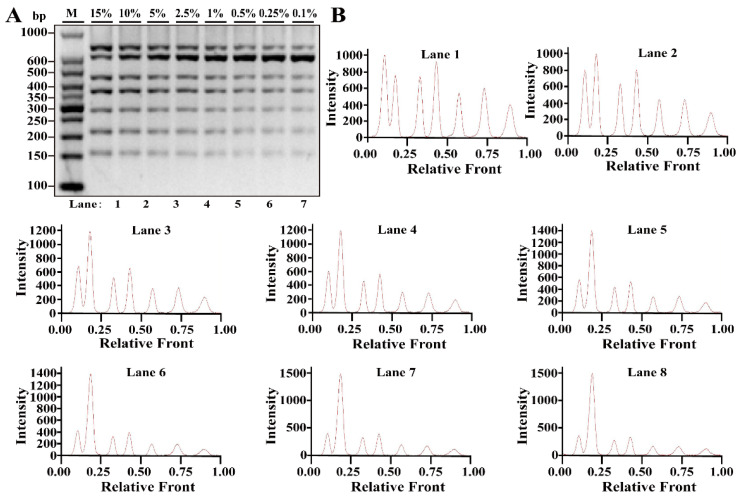
Validation of sensitivity of multiplex PCR assay in meat model mixtures. (**A**) Gel image of PCR fragments amplified by multiplex PCR using DNA from model mixtures of camel, dog, pig, chicken, sheep and duck added to cattle at 15%, 10%, 5%, 2.5%, 1%, 0.5%, 0.25% and 0.1% of total weight, respectively. (**B**) The corresponding electropherograms represent camel, cattle, dog, pig, chicken, sheep and duck in each lane. Lanes 1–8 are presented with labels (15%, 10%, 5%, 2.5%, 1%, 0.5%, 0.25% and 0.1%) in (**A**). The value of number at the horizontal line means the relative position of peaks distant from the top of agarose gel. The value of number at the vertical line means the fluorescent intensity of DNA-bound dyes (4 S GelRed Nucleic Acid Stain). Lane M is ladder DNA.

Differing from simplex PCR assays, multiplex PCRs are complicated with the increasing multiplicity of PCR reaction caused by increased primer pairs. Based on simplex PCR system, multiplex PCR systems starting from duplex, triplex, tetraplex, pentaplex and hexaplex PCRs were gradually developed and a heptaplex PCR method was ultimately constructed by seven sets of target primers. To examine the limit of detection (LOD) and dynamic range of this method, multiplex PCR assays were performed using the serial DNA concentration of each species. As shown in Figure 2A, seven bands corresponding to seven species were clearly visible at concentrations ranging from 10 to 0.025 ng DNA, while 0.01 ng DNA of some species generated relatively weak bands. According to the bands, electropherograms were drawn using Image Lab^TM^ Software, in which the intensities of the peak patterns were associated with the brightness of the bands. With decreasing DNA concentrations, the intensities of peaks were obviously reduced showing a severe defect of peak pattern at 0.01 ng DNA (Figure 2B). Therefore, the LOD of this heptaplex PCR was defined as 0.025 ng DNA for the simultaneous identification of seven species. In addition, model cattle mixed with six meat tissues of camel, dog, pig, chicken, sheep and duck at 0.1%, 0.25%, 0.5%, 1%, 2.5%, 5%, 10% or 15% of the total weight were individually prepared and used for LOD analysis. As shown in Figure 3A,B, the specific amplicons for all target species were availably detected even at the target meat percentages of 0.1%, further supporting the opinion that this method is adequate for meat inspection.

### 3.3. Reproducibility of Multiplex PCR Assay in Heat Processing Meat

**Figure 4:**
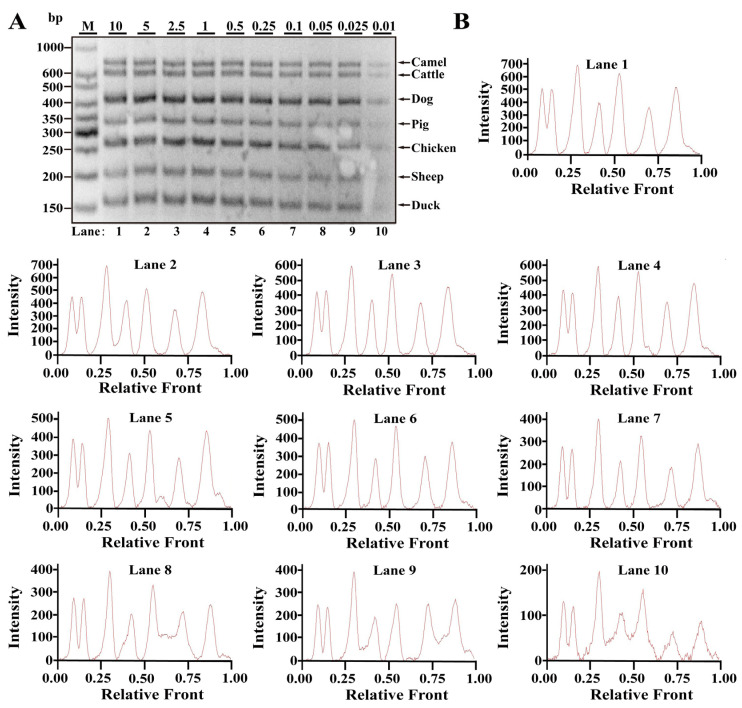
Validation of sensitivity of multiplex PCR assay in boiling meat tissues. (**A**) Gel image of PCR fragments amplified by multiplex PCR using premixed DNA contents of seven species (10, 5, 2.5, 1, 0.5, 0.25, 0.1, 0.05, 0.025 and 0.01 ng) with seven sets of target primers, respectively. (**B**) The corresponding electropherograms represent camel, cattle, dog, pig, chicken, sheep and duck in each lane. Lanes 1–10 are presented with labels (10, 5, 2.5, 1, 0.5, 0.25, 0.1, 0.05, 0.025 and 0.01) in (**A**). The value of number at the horizontal line means the relative position of peaks distant from the top of agarose gel. The value of number at the vertical line means the fluorescent intensity of DNA-bound dyes (4S GelRed Nucleic Acid Stain). Lane M is ladder DNA.

**Figure 5:**
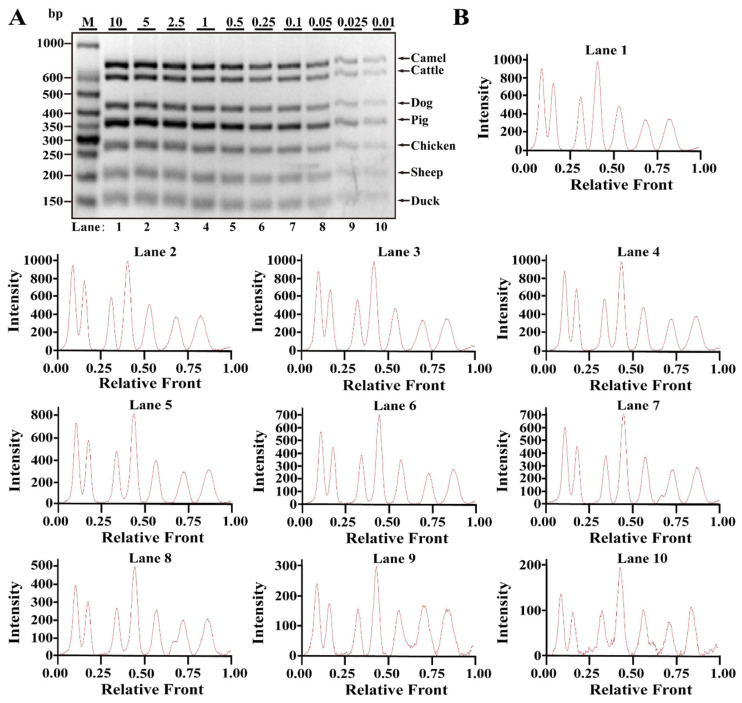
Validation of sensitivity of multiplex PCR assay in microwave-cooking meat tissues. (**A**) Gel image of PCR fragments amplified by multiplex PCR using premixed DNA contents of seven species (10, 5, 2.5, 1, 0.5, 0.25, 0.1, 0.05, 0.025 and 0.01 ng) with seven sets of target primers, respectively. (**B**) The corresponding electropherograms represent camel, cattle, dog, pig, chicken, sheep and duck in each lane. Lanes 1–10 are presented with labels (10, 5, 2.5, 1, 0.5, 0.25, 0.1, 0.05, 0.025 and 0.01) in (**A**). The value of number at the horizontal line means the relative position of peaks distant from the top of agarose gel. The value of number at the vertical line means the fluorescent intensity of DNA-bound dyes (4 S GelRed Nucleic Acid Stain). Lane M is ladder DNA.

To determine the practicability of the technique in thermally processed meat, raw meat tissues were, respectively, subjected to boiled and microwave-cooked treatments. For boiling meat samples, genomic DNA was isolated from meat tissue exposed to a heat processing treatment of boiled patterns (97–99 °C, 30 min). Multiplex PCR assays showed that seven bands for seven meat species were clearly observed at the range of 0.025–10 ng DNA and intact peak patterns for seven species were obtained in lanes 1–9 (Figure 4A,B), suggesting that the LOD of the heptaplex PCR method was 0.025 ng DNA for seven boiling meat tissues. Meanwhile, microwave-cooked meat tissues were used for DNA isolation, and then, multiplex PCR assays were conducted. Differing from boiling meat tissues, microwave-cooked meat tissues showed that the LOD was approximately 0.01 ng DNA for all meat species (Figure 5A,B). Overall, it is postulated that the threshold value was about 0.01–0.025 ng DNA for the discrimination of heat-processed meat. The data further demonstrate the availability of this method for the inspection of animal species in real-world meat products.

### 3.4. Application of Multiplex PCR Assay on Commercial Meat Products

To validate the practicability of this method on the authentication of commercial meat products, popular consumption products such as drysaltery, jerky, meatballs, meat slices and kebab were selected to assess meat authenticity. As summarized in Table 2, most food items had the same meat materials as declared. However, some samples contained extra ingredients that were unlabeled: 5 of 15 (33.3%) sheep samples, 4 of 15 (26.7%) cattle samples and 1 of 10 (10%) camel samples that were declared to be 100% pure meat contained unlisted meat adulterants. In addition, this survey revealed that poultry derivatives, as adulterants are frequently adulterated into real-world meat products.

## 4. Discussion

Adulteration practice has been cleverly conducted leading to escape from visual detection since they show morphological and physical characteristics similar to pure meat [5]. The DNA-based techniques of PCR are recognized as the most available methods applied to species authentication in various pattern meats [2]. Compared to conventional PCR, multiplex PCR systems are greatly auspicious as they can discriminate multiple meat types in a single platform through simple agarose gel analysis and show a dramatically reduced cost and time of analysis [25,26,27]. Using multiplex PCRs, it is complicated to optimize the same conditions for multiple sets of primers in the same PCR reaction, because the mutual interference of the components becomes more complex with the increase in the number of primers and multiplicity of multiplex PCR reactions [6]. Therefore, they are often subjected to technological challenges. Through the survey of multiplex PCRs recently published in Table 3, most multiplex PCRs discriminate less than eight meat species in one reaction platform. Although three studies have authenticated more than ten animal species, they are all achieved by two-tube multiplex PCR assays [6,24,28]. Here, this proposed method can examine seven meat origins by seven sets of highly specific primers. Most importantly, LOD of multiplex PCR assays vary from 1 pg to 0.5 ng or 0.01% to 9.1% (*w*/*w*) of the total meat weight (Table 3), while the LOD of this study is about 0.025 ng DNA or target meat of 0.1% (*w*/*w*) of the total meat weight in various meat samples, including boiled and microwave-cooked meat materials, indicating that this method would have a good application in real-word meat inspection.

In this study, all meat species were selected based on the works in the published literature, as listed in Table 3. In fact, most of meat species, such as cattle, pig, chicken, sheep and duck, were considered based on actual adulteration cases with a higher practicability in Chinese markets. Consistent with other studies [6,9], this survey reveals that meat adulteration with inferior ingredients such as chicken, duck and pork are still rampant in the Chinese market, indicating that it is of great importance to develop practical techniques to assess meat authenticity. PCR assays occasionally cause artifacts due to contamination by alien DNA and generate non-specific target amplification [40]. To ensure the reliability of PCR authentication, it is highly necessary to analyze the amplicons of each target species by DNA sequencing. To confirm the specific amplification of target primers, a DNA mixture of seven species was used for PCR amplification. The recombinant plasmids were individually constructed by connecting PCR products into a cloning vector, which were further used as a template for PCR amplification. DNA sequencing demonstrated the specific PCR amplification of the target species with an accuracy of 100% based on BLAST analysis and comparison against the NCBI database. Most importantly, this multiplex PCR system can capture the template DNA from heat-processed meat samples and generate the expected PCR fragments. Overall, it can be speculated that the robust reproducibility of target primers guarantees the accuracy of this method when applied to commercial meat products.

## 5. Conclusions

The validation of meat authenticity is crucial to safeguard consumers from food fraud and thereby establish discipline in the food industry. In this study, the proposed heptaplex PCR method is a reliable, low-cost and rapid approach for the simultaneous discrimination of camel, cattle, dog, pig, chicken, sheep and duck. Especially, DNA sequencing demonstrated that target primers can specifically amplify target fragments from a DNA mixture of seven species. The technique was availably applied to various processing meat samples and was found to provide an accurate assessment of commercial meat products. In summary, this study develops a powerful tool for supervising food quality and protecting consumers from meat deception.

## Figures and Tables

**Table 1:** Oligonucleotide primers for meat species used in this study.

Primers	Genes	Sequence (5′-3′ Direction)	Amplicons (bp)	Reference or Source
Camel	NADH dehydrogenase subunit 4	TCGCCAGCCAATCTCACCTCT	726	this study
		CATGGGCAACTATAAGGGTCGTA		
Cattle	Cytochrome c oxidase subunit I	ATGAGCCCACCATATATTCACT	610	this study
		TGTCGTGGTTAAGTCTACAGTCA		
Dog	NADH dehydrogenase subunit 5	TGGCCTATTTAAAGTCCTTCCCT	428	this study
		CTAGTGCCAGGATGAAACCCAA		
Pig	NADH dehydrogenase subunit 4	AGCCTATCCATTCCTCATGCTTT	332	this study
		CTAGGTTTGTGAGGCTTGCTAC		
Chicken	16S rRNA	GAGTGCGTCAAAGCTCCCTC	268	this study
		GTTTGCCGAGTTCCTTCTGT		
Sheep	NADH dehydrogenase subunit 2	ATCCAATAGCCTCCATACTCA	205	this study
		ATTGATAGGGTTAGGATCAGGTC		
Duck	Cytochrome c oxidase subunit III	TCCACGCCCTAACATTGACGATT	163	this study
		AAGGTGGATCCGATGATCACT		
Eukaryotes	12S rRNA	CAACTGGGATTAGATACCCCACTAT	456	[20]
		GAGGGTGACGGGCGGTGTGT		
Eukaryotes	16S rRNA	AAGACGAGAAGACCCTATGGA	240	[21]
		GATTGCGCTGTTATCCCTAGGGTA		
Eukaryotes	18S rRNA	AGGATCCATTGGAGGGCAAGT	99	[22]
		TCCAACTACGAGCTTTTTAACTGCA		

**Table 2:** Results of multiplex PCR assay performed on commercial meat products.

Products	Number	Labelled	Detected Species							Adulteration
			Camel	Cattle	Dog	Pig	Chicken	Sheep	Duck	
Sheep	15									5 (33.3%)
meat balls	5	mutton				1/5 ^a^, 1/5 ^b^	1/5 ^a^	5/5	1/5 ^b^	
meat slices	5	mutton				1/5		5/5		
kebab	5	mutton				2/5		5/5		
Cattle	15									4 (26.7%)
meat balls		beef		5/5		1/5 ^a^, 1/5 ^b^	1/5 ^c^		1/5 ^a^	
meat slices		beef		5/5						
kebab		beef		5/5		1/5				
Camel	10									1 (10%)
drysaltery	5	camel	5/5			1/5				
jerky	5	camel	5/5							

In each row, meat samples labeled with same letter (a, b or c) represent the identical meat samples, whereas different letters indicate a difference in meat samples.

**Table 3:** Comparative analysis of multiplex assays for the identification of species origin of meat.

Multiplex PCR Type	Species Number	Detection Items	Detection Limit	Detection Method	Reference or Source
Heptaplex	7	camel, cattle, dog, pig, chicken, sheep and duck	0.025 ng DNA or 0.1% for each species	Gel	This study
Multiplex	3	chicken, turkey, duck	1 pg for each species	Gel	[29]
Tetraplex	3	pig, cattle, fish	0.001–0.1 ng DNA	Gel	[30]
Multiplex	4	ruminant, poultry, pork, donkey	0.01–0.1 ng/μL DNA	Gel	[1]
Multiplex	4	pork, chicken, duck, cattle	0.1% for each species	Gel	[31]
tetraplex	4	horse, soybean, poultry, pork	0.01% for each species	Gel	[32]
Multiplex	4	chicken, duck, pork, beef	0.05% for each species	Gel	[33]
Multiplex	4	buffalo, cattle, pork, duck	1 pg DNA, 0.1% for each species	Gel	[34]
Quadruplex	4	chicken, mutton, beef, pork	16 pg DNA, 0.01% of each species	Gel	[35]
Multiplex	4	rat, fox, duck, sheep	0.05 ng/μL DNA	Gel	[36]
Multiplex	5	cat, dog, pig, monkey, rat	0.01–0.02 ng DNA	chip	[19]
Multiplex	5	sheep/goat, bovine, chicken, duck, pig	0.5 ng DNA	Gel	[26]
Hexaplex	6	horse, soybean, sheep, poultry, pork, cow	0.01% for each species	Gel	[37]
Hexaplex	6	chicken, cow/buffalo, sheep/goat, horse/donkey, pork, dog	0.03–0.05 ng DNA	Gel	[25]
Multiplex	6	goat, chicken, cattle, sheep, pig, horse	0.25 ng DNA	Gel	[38]
Multiplex	6	mutton, pork, duck, chicken, horse, cat	9.1% of each species	Gel	[39]
Septuple	7	turkey, goose, pig, sheep, beef, chicken, duck	0.01–0.05 ng DNA	Gel	[17]
Heptaplex	7	pig, beef, horse, duck, chicken, pigeon, camel	0.01–0.025 ng DNA or 0.1% for each species	Gel	[3]
Octuplex	8	dog, chicken, cattle, pig, horse, donkey, fox, rabbit	0.05 ng/μL DNA	Gel	[21]
Multiplex (two-tube)	10	beef, sheep, pork, chicken, turkey; cat, dog, mouse, rat, human	30 pg DNA	Gel	[24]
Multiplex (two-tube)	12	horse, pigeon, camel, rabbit, ostrich, beef; turkey, dog, chicken, duck, cat, goose	0.05–0.1 ng DNA	Gel	[28]
Multiplex (two-tube)	14	cattle, donkey, canidae (dog, fox, raccoon-dog), deer, horse; pig, ovis (sheep, goat), poultry (chicken, duck), cat, mouse	0.02–0.2 ng DNA	Chip	[6]

Chip, microchip electrophoresis; Gel, agarose gel electrophoresis.

## Data Availability

The original contributions presented in the study are included in the article; further inquiries can be directed to the corresponding author/s.
